# Association between miR-27a genetic variants and susceptibility to colorectal cancer

**DOI:** 10.1186/1746-1596-9-146

**Published:** 2014-07-30

**Authors:** Zaiqiu Wang, Xiaoli Sun, Yeli Wang, Xiaofang Liu, Yuanjie Xuan, Sanyuan Hu

**Affiliations:** Department of General Surgery, Qilu Hospital of Shandong University, Jinan, Shandong 250012 China; Department of Anorectal, Yuhuangding Hospital, Yantai, Shandong 264000 China; Department of Laboratory, Yuhuangding Hospital, Yantai, Shandong 264000 China; Department of Hepatobiliary, Yuhuangding Hospital, Yantai, Shandong 264000 China

**Keywords:** Single nucleotide polymorphism, Colorectal cancer, miR-27a, Risk factor

## Abstract

**Background:**

MicroRNAs (miRNAs) are short, non-coding RNAs that negatively regulate target genes. A single nucleotide polymorphism (SNP) in a miRNA sequence may alter miRNA expression and/or maturation, which was proposed to associate with the development and progression of cancer. The rs895819 polymorphism, located in the terminal loop of pre-miR-27a, has been reported to have relevance to several cancers. In this study, we investigated the possibility of association between polymorphism in rs895819 and susceptibility to colorectal cancer (CRC).

**Methods:**

We identified a single SNP, rs895819 in pre-miR-27a, for further investigation, were determined in 205 CRC patients and 455 healthy controls.

**Results:**

When taking the AA genotype as a reference, we found that AG genotype was not statistically significantly associated with the risk of CRC (AG vs. AA, OR 1.245, 95% CI: 0.806 – 1.923). However, the GG genotype was significantly associated with risk of CRC (GG vs. AA, OR 1.599, 95% CI: 1.052 – 2.430). In the AG + GG vs GG group, no significant difference was detected (OR 1.424, 95% CI, 0.974 – 1.801). GG genotype and G allele was associated with an increased risk of metastasis in this study (P < 0.001 and P = 0.003, respectively).

**Conclusions:**

This study found significant association between rs895819 polymorphism in pre-miR-27a and CRC risk. Population-based studies with large number of subjects and long-term follow-up are needed to verify the association of miR-27a polymorphism with CRC susceptibility and severity.

**Virtual Slides:**

The virtual slide(s) for this article can be found here: http://www.diagnosticpathology.diagnomx.eu/vs/2061490734125077

## Background

Colorectal cancer (CRC) is reported to be the third most common cancer worldwide with an estimated one million new cases and it is one of the most frequent cancers and a common cause of cancer-related deaths [1–3]. Improvements of diagnosis and treatment had resulted in improved long-term survival rates for patients with early CRC, whereas the outlook for individuals with advanced disease remains poor. Advanced CRC frequently recurs as nodal and hematogenous metastasis and peritoneal dissemination [4]. Although several types of non-surgical treatments have been assessed, surgery is the primary treatment for CRC, and if the tumor is caught at a sufficiently early stage, surgery can be curable. The nature of the disease is, however, such that it often presents at an advanced stage, and so surgery alone is insufficient [5].

CRC development involves a multi-step process including both genetic and epigenetic changes, which leads to activation of oncogenes and inactivation of tumor suppressor genes in cancer cells [6]. MicroRNAs (miRNAs) are short, non-coding RNAs of approximately 23 nucleotides that negatively regulate target genes. MiRNAs have been implicated in several biochemical pathways in the eukaryotic cells of various organisms [7]. As a posttranscriptional regulator, miRNAs bind to complementary sequences on target messenger RNAs (mRNAs), resulting in mRNA degradation or translational repression. Sequences encoding the miRNA precursor are usually transcribed by RNA polymerase II to generate primary miRNAs (pri-miRNAs). A strong association between miRNA and cancer has been recently reported. MiRNAs have been proposed to contribute to tumorigenesis as either tumor suppressors or oncogenes [8]. A single nucleotide polymorphism (SNP) is a single nucleotide variation of a DNA sequence, A (adenine), T (thymine), C (cytosine), or G (guanine), on genomic DNA. A SNP in a miRNA sequence may alter miRNA expression and/or maturation and be associated with the development and progression of cancer [9].

The relationship between miR-27a and several different cancers have been discussed. Tang et al. reported that the expression of miR-27a was significantly correlated with clinic pathological parameters, including tumor size, lymph node metastasis and distant metastasis, but not with receptor status [10]. Patients with high miR-27a level tended to have significantly shorter disease-free survival durations and overall survival times [10]. It was also reported that the expression of miR-27a was higher in human ovarian cancer relative to benign ovarian tissues. Transfection of SKOV3 cells with the miR-27a inhibitor could suppress the growth and migration of tumor cells [11].

We utilized public databases to identify deregulated miRNAs in CRC and SNPs in these miRNAs sequences, including primary, precursor and mature miRNAs. Our searches identified a single SNP, rs895819 in pre-miR-27a, for further investigation. The rs895819 polymorphism is located in the terminal loop of pre-miR-27a and involves an AG nucleotide transition. The association between rs895819 polymorphism and risk of several cancers were reported [12–14]. In this study, we investigated the possibility of association between polymorphism in rs895819 and susceptibility to CRC.

## Methods

### Ethics statement

This study was approved by the institutional review board of Qilu Hospital of Shandong University. All the participants were voluntary and provided written informed consent prior to taking part in this research.

### Study subjects

All subjects were recruited from Qilu Hospital of Shandong University between January 2010 and December 2013. They were all newly diagnosed, histopathologically confirmed and without a prior history of cancer or previous chemo- or radiotherapy. In all, 205 patients with CRC were recruited, all of whom were unrelated ethnic Han Chinese population. A structured questionnaire on demographics and environmental exposure, including age, sex, smoking consumption and alcohol drinking, was conducted by trained interviewers through face-to-face interviews with the patients. In addition, 5 ml venous blood was collected from each patient for genomic DNA extraction. The clinical characters of all cases were recorded by reviewing the databases or counseling the doctor.

### DNA extraction

For both the cases and control cohorts, 1.5 ml of whole blood was extracted from each participant and stored at −80°C before being used. DNA from each sample of whole blood was extracted with the QIAamp DNA mini Kit (Qiagen, Hilden, Germany), as directed by the manufacturer's instructions. The concentration of DNA and the purity of each sample were measured by an ultraviolet spectrophotometer (GE Healthcare, USA). DNA samples were routinely stored at − 20°C.

### SNP genotyping analysis

Genotyping was performed by TaqMan allelic discrimination assays using an ABI 7900 system (Applied Biosystems, Foster City, CA, USA). The prime and probes are as follows: forward, GGCGGAACTTAGCCACTGT, reverse, CAGGGCTTAGCTGCTTGTG. The TaqMan assays were performed in a final reaction volume of 5 ml containing 0.25 ml primer, 0.125 ml probe, 2 ml PCR mixture reagent and 25 ng DNA. The PCR reaction consisted of an initial step at 95 uC for 10 min followed by 55 cycles of denaturing at 95 uC for 15 s and annealing at 60 uC for 60 s. SDS allelic discrimination software (ABI) was used to analyze the PCR genotyping results. Two blank (water) controls were included in each 384-well assay. At least 10% of samples were randomly selected for repeat analysis, yielding 100% concordance. A further 60 samples were selected randomly for direct sequencing to confirm the TaqMan results. Again, the results showed 100% concordance.

### Statistical analysis

Statistical analysis was performed using the SPSS 20.0 software package (SPSS Company, Chicago, IL). Hardy–Weinberg equilibrium analysis was performed using a goodness-of-fit χ^2^ -test with one degree of freedom. P < 0.05 was considered representative of a departure from HWE. Comparison of allele type and genotype distributions in the patients and healthy controls were performed by means of two-sided contingency tables using the χ^2^ -test. The odds ratio (OR) and 95% confidence interval (CI) for genotypic-specific risk were calculated using an unconditional logistic regression model. P < 0.05 was considered significant for all statistical analyses.

## Result

### General characteristics of the subjects

A total of 205 CRC patients and 455 healthy controls were enrolled in our study. All of the subjects were ethnic Han Chinese. Demographic and other selected characteristics of cases and controls were presented in Table 1. Cases and controls did not show statistically significant differences with regard to sex and age. However, the higher smoking and alcohol drinking were risks of CRC (P = 0.001, OR = 1.868; P = 0.044, OR = 1.405, respectively). When the duration or amount was considered, higher amount (OR, 2.34, 95% CI, 1.58 -3.46) and duration (OR, 2.43; 95% CI, 1.389 – 3.005) of tobacco smoking were associated with the risk of CRC. Besides, the higher amount of alcohol consumption was associated with higher risk of CRC (OR, 1.790; 95% CI, 1.230 – 2.606). In the advanced analyses, the results showed that high intake of vegetable (OR, 0.310; 95% CI, 0.202 – 0.476) and fruit intake (OR, 0.588; 95% CI, 0.380 – 0.899) was associated with reduced risk of CRC incidence. The education statuses were not associated with the risk of CRC, while lower income might lead to high incidence of CRC (OR, 0.252; 95% CI, 0.143 – 0.444).

**Table 1:** **General characteristics of colorectal cancer (CRC) patients and controls**

Variable s	Cases	Percentage (%)	Control	Pecentage (%)	P value	OR (95% CI)
Age (years, mean ± SD)	54.9 ± 11.2		55.4 ± 10.1		0.15	
Sex						
Male	103	50.24	225	49.45		1 (Ref)
Female	102	49.76	230	50.55	0.859	1.032 (0.742 - 1.435)
Smoking status						
No	61	29.76	201	44.18		1 (Ref)
Yes	144	70.24	254	55.82	< 0.001	**1.868 (1.314 - 2.656)**
Smoking amount (pack-years)						
0	61	29.76	201	44.18		1 1 (Ref)
< 1500	51	24.87	123	27.03	0.453	1.21 (0.88 – 2.11)
> 1500	93	45.36	131	28.79	< 0.001	**2.34 (1.58 -3.46)**
Smoking duration (years)						
0	61	29.76	201	44.18		(Ref) 1
< 5	12	5.85	37	8.13	0.325	1.069 (0.525 – 2.177)
5 - 10	39	19.02	81	17.80	0.067	1.587 (0.984 – 2.558)
> 10	93	45.36	150	32.97	0.002	**2.43 (1.389 – 3.005)**
Alcohol						
No	89	43.41	236	51.87		1 (Ref)
Yes	116	56.59	219	48.13	0.044	**1.405 (1.008 - 1.957)**
Alcohol amount (cups/day)						
0	89	43.41	236	51.87		1 (Ref)
1 – 3	39	19.02	102	22.42	0.284	1.014 (0.651 – 1.578)–
> 3	77	38.54	117	26.71	0.012	**1.790 (1.230 – 2.606)**
Alcohol use duration						
0	89	43.41	236	51.87		1 (Ref)
< 5	32	15.61	78	17.53	0.329	1.088 (0.674 – 1.755)
5 - 10	29	14.15	65	14.61	0.436	1.183 (0.717 – 1.953)
> 10	55	26.83	76	17.08	< 0.001	1.919 (1.256 – 2.933)
Fruit intake (times/week)						
≤ 1	68	33.17	120	26.97		1 (Ref)
2-4	53	25.85	165	37.07	0.042	0.588 (0.380 – 0.899)
≥ 5	84	40.98	170	38.20	0.245	0.872 (0.587 – 1.296)
Vegetable intake (times/week)						
≤ 1	85	41.46	116	26.07		1 (Ref)
2-4	75	36.59	131	29.43	0.546	0.781 (0.524 – 1.164)
≥ 5	45	21.95	198	44.49	< 0.001	**0.310 (0.202 – 0.476)**
Education status						
High school or less	109		228			1 (Ref)
College or University	81		202			0.839 (0.594 – 1.183)
Master or more	15		15			2.092 (0.987 – 4.434)
Income (RMB/year)						
≤ 100,000	98	47.80	109	24.49		1 (Ref)
100,000 – 300,000	88	42.93	252	56.12	0.001	0.388 (0.270 – 0.560)
≥300,000	19	9.27	84	18.88	< 0.001	0.252 (0.143 – 0.444)

With regard to clinic pathologic characteristics of CRC patients, most patients had a smaller tumor size (less than 5 cm, 65.85%). Well-moderately status was in 126 (61.46%) patients, while poorly-undifferentiated status was in 79 (38.54%) patients. A total of 86 patients (41.95%) had metastasis among all the patients. According to the TNM classification criteria, 138 and 67 patients were in stage I-II and III-IV, respectively (Table 2).

**Table 2:** **Clinical characteristics of colorectal cancer (CRC) patients and controls**

Variables	Cases	Percentage (%)
Tumor size		
<5 cm	135	65.85
≥5 cm	70	34.15
Differentiated status		
Well-moderately	126	61.46
Poorly-Undifferentiated	79	38.54
TNM stage		
I-II	138	67.32
III- IV	67	32.68
Metastasis		
Yes	86	41.95
No	119	58.05
Site		
Colon	108	52.68
Rectum	73	35.61
Both	24	11.71
Therapy		
Sugery	126	61.46
Local resection	23	11.21
Chemotherapy	56	27.31

### The effect of the rs895819 polymorphism on the risk of CRC

The genotype and allele frequencies of the rs895819 were presented in Table 3. The genotype frequencies in both cases and controls were in agreement with the HWE model. As shown in Table 3, the genotype frequencies of the rs895819 were 11.71, 39.02 and 49.27% for the AA, AG, and GG genotypes respectively, among the CRC group; and 30.33, 34.51 and 35.16% among the controls, respectively. When taking the AA genotype as a reference, we found that AG genotype was not statistically significantly associated with the risk of CRC (AG vs. AA, OR 1.245, 95% CI: 0.806 – 1.923). However, compared with AA genotype, the GG genotype was significantly associated with risk of CRC (GG vs. AA, OR 1.599, 95% CI: 1.052 – 2.430). In the AG + GG vs GG group, no significant difference was detected (OR 1.424, 95% CI, 0.974 – 1.801).

**Table 3:** **Genotypes of rs895819 polymorphism and CRC risk**

Genotype	Cases	Percentage (%)	Control	Pecentage (%)	P value	OR (95% CI)
AA	48	11.71	138	30.33		1 (Ref)
AG	68	39.02	157	34.51	0.087	1.245 (0.806 – 1.923)
GG	89	49.27	160	35.16	0.015	**1.599 (1.052 – 2.430)**
AG + GG	157	88.29	317	69.67	0.064	1.424 (0.974 – 1.801)
Allele frequency						
A allele	164	32.22	433	49.58		1 (Ref)
G allele	246	67.78	477	50.42	0.027	**1.362 (1.075 – 1.725)**

Besides, the allele distribution was significant difference between cases and controls (OR 1.362, 95% CI 1.075 - 1.725). Taken together, these data suggest that the miR-27a binding site polymorphism rs895819 may be associated with the risk of CRC.

### The rs895819 polymorphism and clinicopathological characteristics

In the stratified studies, we attended to detect the association between rs895819 polymorphism and clinicopathological characteristics of patients with CRC. We observed no association between rs895819 polymorphism and the progression of CRC including tumor size and tumor stage. However, GG genotype and G allele was associated with an increased risk of metastasis in this study (P < 0.001 and P = 0.003, respectively) (Table 4).

**Table 4:** **Clinicopathological characteristics and miR-27a genotype and allelic frequencies of CRC patients**

	Genotype			P value		Allele		
	AA	AG	GG	AG vs AA	GG vs AA	A	G	P value
Tumor size								
<5 cm	33	40	62			103	162	
≥5 cm	16	20	34	0.784	0.812	52	88	0.734
TNM stage								
I-II	32	43	60			105	163	
III- IV	15	21	34	0.729	0.617	51	89	0.587
Metastasis								
Yes	19	20	47			58	114	
No	28	31	69	0.903	**< 0.001**	87	169	**0.003**

## Discussion

According to the polygenic model of carcinogenesis, unfavorable combinations of polymorphic genetic variants in low-penetrance susceptibility genes contribute to increasing CRC risk. Studies have shown that profiles of miRNA expression differ between normal and tumor tissues, which vary among tumor types and the down-regulation of miRNA subsets implies a tumor-suppressor function, which is often observed in tumor development. Recently, a class of novel functional polymorphisms in miRNAs or their binding sites has attracted great interest. One important SNP in pre-mir-27a of an A to G change was identified as rs895819, and its role in cancer susceptibility has been discussed recently [14, 15]. Though many studies have been performed, whether rs895819 contributes to cancer susceptibility is still unclear, and the association between rs895819 and CRC risk remains unknown. Therefore, our present study investigated the association of rs895819 with CRC risk in the Chinese population. In a case–control study with 205 cases and 455 controls, our study demonstrated that subjects with GG genotype and the G allele exhibited a significantly increased risk of CRC in the cohort. Advanced study on the association between rs895819 and the clinical pathological characters showed that GG genotype and the G allele were risk factors of increased risk of CRC.

A previous study by Fu et al. identified 32 differentially expressed miRNAs and 2916 mRNAs from CRC samples and their corresponding normal epithelial tissues by miRNA and mRNA microarray, respectively. Additionally, 6 up-regulated miRNAs (mir-21, mir-223, mir-224, mir-29a, mir-29b, and mir-27a) and 4 down-regulated predicted target mRNAs (SFRP1, SFRP2, RNF138, and KLF4) were selected to validate the expression level and their anti-correlationship in an extended cohort of CRC patients by qRT-PCR [16]. In animal model, it was also reported that a recurrent amplicon on mouse chromosome 8 that encodesmiR-23a and -27a levels are up-regulated in mouse intestinal adenocarcinomas, primary tumors from patients with stage I/II colorectal cancers, as well as in human colorectal cancer cell lines and cancer stem cells [11]. It could be presumed that miR-27a might play a key role in the development and progression of CRC. Some more studies were also conducted to identify the potential role of miR-27a in CRC. Kim et al. showed that human miR-27a* is a negative regulator of NK-cell cytotoxicity by silencing Prf1 and GzmB expression. Human miR-27a* specifically bound to the 3' untranslated regions of Prf1 and GzmB, down-regulating expression in both resting and activated NK cells, and it functioned as a fine-tuner for homeostasis of the net amount of the effectors proteins. Consistent with miR-27a* having an inhibitory role, knockdown of miR-27a* in NK cells dramatically increased cytotoxicity in vitro and decreased tumor growth in a human tumor xenograft model. Thus, NK-cell cytotoxicity is regulated, in part, by microRNA, and modulating endogenous miR-27a* levels in NK cells represents a potential immunotherapeutic strategy [17]. Resveratrol and quercetin (RQ) in combination (1:1 ratio) could produce a significant anticancer effect through decreasing the miR-27a level in colon cancer cell lines [18]. This was confirmed by transfection of cells with the specific mimic for miR-27a, which partially reversed the effects of RQ.

Single nucleotide polymorphism of miRNA would influence the expression of miRNA and thus would influence the cancer incidence or progression. Zhou et al. [19] reported that 19 SNPs of the miRNA-related genes in a case–control study of 311 gastric cancers and 425 cancer-free controls in a Chinese Han population. The results showed that the SNP rs895819 in the miR-27a gene with the minor allele C presented significantly reduced risk to gastric cancer (p = 0.037, OR = 0.771, 95% CI = 0.604 - 0.985). Wang et al. [20] conducted a meta-analysis of miR-27a SNP and risk of breast cancer. They found that hsa-mir-27a rs895819 polymorphism also did not reveal any relationship with breast cancer susceptibility, while significantly decreased risk was found among Europeans in AG versus AA and AG/GG versus AA models tested (AG versus AA: OR = 0.83; 95% CI, 0.72-0.97; GG versus AA: OR = 0.86; 95% CI, 0.71-1.05; AG/GG versus AA: OR = 0.84; 95% CI, 0.75-0.94).

The association between miR-27a SNP and CRC risk was also studied in several studies. Hezova R et al. [21] conducted a case–control study to investigate whether selected single nucleotide polymorphisms (SNPs) in miR-196a2, miR-27a and miR-146a genes were associated with sporadic colorectal cancer (CRC). The results of this case–control study of 197 cases of sporadic CRC and 212 cancer-free controls originating from the Central-European Caucasian population showed that there was a lack of association between rs11614913, rs895819 and rs2910164 and colorectal cancer risk in the Central-European Caucasian population. This result was contradictory with our study. The potential explanations were the difference between the locality and races included in the two studies. The genetic background and environmental factors might influence the effect of rs895819 on the risk of CRC.

## Conclusion

Our results showed an association between miR-27a polymorphism and CRC in Chinese Han patients. Besides, miR-27a polymorphism showed a significant association with the risk of metastasis. However, no functional validation of these observational findings was conducted and a series of following studies would be conducted. Considering that this is from a case-control study, however, population-based studies with large number of subjects and long-term follow-up are needed to verify the association of miR-27a polymorphism with CRC susceptibility and severity.

## Authors’ information

Zaiqiu Wang and Xiaoli Sun are co-first authors.
